# Anti-Tr/DNER antibody paraneoplastic cerebellar degeneration preceding a very late relapse of Hodgkin Lymphoma after 12 years

**DOI:** 10.1186/s40673-021-00137-1

**Published:** 2021-06-05

**Authors:** Peter Broegger Christensen, Henrik Gregersen, Charlotte Almasi

**Affiliations:** 1 Department of Neurology, Aalborg University Hospital, Ladegaardsgade 5, 9000 Aalborg, Denmark; 2 Department of Hematology, Aalborg University Hospital, Aalborg, Denmark; 3 Department of Nuclear Medicine, Aalborg University Hospital, Aalborg, Denmark

**Keywords:** paraneoplastic syndrome, cerebellar degeneration, Hodgkin lymphoma, relapse

## Abstract

**Background:**

Paraneoplastic cerebellar degeneration (PCD) is a classic neurological syndrome where the presence of Anti-Tr/DNER antibodies is strongly associated with Hodgkin Lymphoma (HL). Awareness of the syndrome is important because with prompt treatment the prognosis of HL is good. The diagnosis can be a challenge in some patients. The importance of PCD in the detection of a cancer relapse is not clarified. We report the case of a 76-year-old man where a PCD, initially misdiagnosed as a stroke led to a diagnosis of a very late relapse of HL after 12 years.

**Case presentation:**

A 76-year-old male with a 3-week history of unstable walking, slow speech and dizziness was admitted to our stroke unit apparently because the symptoms started acutely. With a diagnostic delay of 3–4 weeks a correct diagnosis of relapse HL was made based on cerebrospinal fluid changes with a strong positive reaction to anti-Tr/DNER antibodies, FDG-PET/CT scan, and biopsy findings. The medical history revealed that the patient had been diagnosed with HL previously, but has been in complete remission for 12 years. The patient was treated with intravenous immunoglobulin, chemo- and radiation therapy. Over the following 6–8 weeks he improved.

**Conclusions:**

Late relapse in HL is very rare. If it occurs it presents as a symptomatic lymphadenopathy. Our case shows, that PCD can be the only presenting symptom of a very late relapse of HL. Paraneoplastic neurological syndromes (PNS) should be considered even in patients with very long cancer remission. PCD can in rare cases mimic a stroke within the posterior circulation. If MR imaging in severe acute/subacute cerebellar syndrome is normal further investigation is mandatory to rule out a PNS, particular in patients with a previous cancer.

## Introduction

Paraneoplastic cerebellar degeneration (PCD) is a classic neurological syndrome associated with well-characterized onconeuronal antibodies and the presence of a cancer. Among several onconeuronal antibodies in PCD, the presence of Anti-Tr/DNER (Delta/notch-like epidermal growth factor-related receptor) is strongly associated with Hodgkin Lymphoma (HL) [1–3]. Normally PCD is recognized by clinical features but in some patients the diagnosis can be a challenge, particularly in those with no active or recent malignancy and in those with an acute onset. Awareness of the syndrome is important because with prompt treatment the prognosis of HL is good with an overall 5-year survival of 87 % [4]. Normally PCD precedes the diagnosis and thereby its presence leads to the discovery of a HL [1, 2], which otherwise had remained undiagnosed. The late relapse rate in HL is only 3.5 % after 5 years and if it occurs it usually presents as symptomatic lymphadenopathy and/or B-symptoms [4]. Opposite, to the well-documented value of early identification of a paraneoplastic neurological syndrome (PNS) in patients with unknown malignancy, the importance of these syndromes in the detection of a cancer relapse is not clarified. We report the case of a 76-year-old man where a PCD, initially misdiagnosed as a stroke led to a diagnosis of a very late relapse of HL after 12 years.

## Case presentation

A 76-year-old male with a 3-week history of unstable walking, slow speech and dizziness was admitted to our stroke unit. An MRI-scan showed no signs of acute ischemia. Further investigations were not performed and he was discharged. The neurological symptoms progressed further over the next 2 weeks and the patient was re-admitted. On examination, he was unable to walk due to truncal ataxia and he had a severe dysarthria. He was alert, without weakness, pyramidal signs, cranial nerve affection or sensory deficits e.g., the neurological signs were purely cerebellar. He denied B-symptoms (fever, night sweats, or unintended weight loss). A repeated MRI-scan was still without structural abnormalities. Routine hematological and biochemical investigations were within normal limits. Cerebrospinal fluid (CSF) examination showed mild pleocytosis, elevation of protein level at 1.12 g/L (range: 0.15–0.50 g/L), normal IgG-index but an oligoclonal band was present. There were no neoplastic cells and flowcytometry was normal. Screening for antibodies to paraneoplastic neurological syndromes was performed by indirect immunofluorescence test (Euroimmun, Lübeck, Germany) and confirmed by using EUROLINE profiles neuronal antigens, 12 recombinant antigens (amphiphysin, CV2, PNMA2 (Ma2/ta), Ri, Yo, Hu, recoverin, SOX1, Titin, Zic4, GAD65 and anti-Tr/DNER (Euroimmun, Lübeck, Germany). The tests showed a strong reaction to anti-Tr/DNER antibodies in both serum and CSF. Immunoreaction against other paraneoplastic antibodies and the mGluR1 and mGluR5 receptor were negative.

**Fig. 1:**
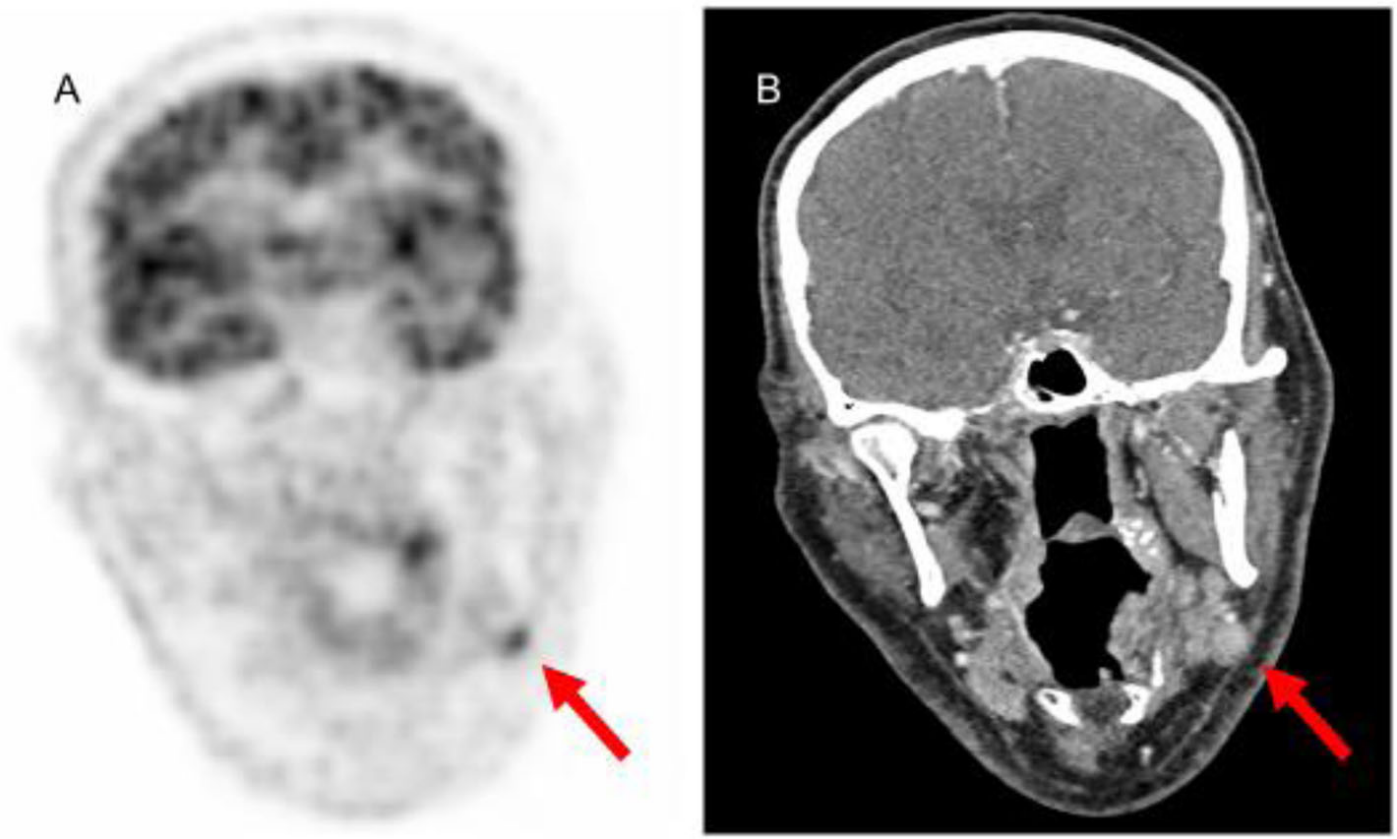
(**A**) Coronal ^18^F-FDG positron emission tomography (PET) and (**B**) contrast enhanced computed tomography (CT). The arrows indicate moderate uptake in a round structure located in close relation to the left submandibular gland. The lesion was new compared to an old ^18^F-FDG PET/CT from 2007 indicating a Warthin tumor or a lymph node. Extirpation/biopsy showed a lymph node with a nodular growth pattern with scattered CD30 and CD 15 positive Reed-Sternberg cells

^18^F-FDG positron emission tomography/contrast enhanced computed tomography (FDG-PET/CT) scan revealed a round structure with moderate FDG uptake in the left submandibular gland (Fig. 1) suggesting a Warthin tumor (lymphomatous papillary cystadenoma). He underwent extirpation of the process which appeared to be a lymph node in close vicinity to the submandibular gland. The node had a thickened lymph nodular capsule and a nodular growth pattern with scattered CD30 and CD15 positive Reed–Sternberg cells. His past medical history revealed that he had been diagnosed with HL previously (Ann Arbor stage III B) but he has been in complete remission for 12 years. Thereafter a diagnosis of relapsed HL was made (Ann Arbor stage I A). As soon as the result of the CSF findings was available, he was treated with intravenous (i.v.) immunoglobulin (IVIG) 0.4 g/kg/day for 5 days followed by 3 additional 5 days i.v. immunoglobulin sequences separated by 4 weeks. The hematological treatment consisted of four 21-day series of i.v. gemcitabine and i.v. liposomal doxorubicin on days 1 and 8 and radiation therapy. The neurological deterioration stopped. Over the following 6–8 weeks he improved. At the last follow-up, 16 months after onset, he could walk assisted by a cane, the dysarthria had improved and he was able to communicate normal. An FDG-PET/CT scan after 12 months showed no sign of HL.

## Discussion

Our case is atypical in several ways. Normally, the PCD precedes the diagnosis of HL in 80 % of the patients [1, 2]. In our patient the recurrence occurred 12 years after a complete remission. Second, HL has a high cure rate and a late relapse is very rare [4]. If it occurs it usually present as symptomatic lymphadenopathy and/or B-symptoms (fever, night sweats, or unintended weight loss) [4]. Presentation of a very late relapse of HL as a PCD has to our knowledge not previously been reported. Third, the neurological outcome in our elderly patient was better than expected. Combining data from the 2 largest series of PCD in HL only 14 % had a full or a partial neurological recovery and patients younger than 40 years were more likely to improve [1, 2]. Fourth, the patient was admitted to your stroke unit where the correct diagnosis initially was not made, although PCD in rare cases can mimic a stroke within the posterior cerebrovascular territory [5].

Opposite, to the well-documented value of early identification of a PNS in patients with unknown malignancy, the importance of these syndromes in the detection of a cancer relapse is unknow and there are limited data on the frequency of PNS in patients with reoccurrence of their malignancy. In a case series of 28 patients with PCD in HL 5 patients had onset of PCD after their lymphoma [2], in 3 of these within the first year. One patient had a mild gait ataxia 10 years after the cure of HL, but no tumor was found after 4 years observation. Delayed onset of a second PNS has been described in 8 patients identified among 979 cases in the PNS Euronetwork database [6]. In 5 of these the second PNS episodes antedated a cancer relapse or a new cancer. In all patients, the second PNS was clinical different from the first, although the antibody was the same, most commonly Anti-Hu. The reason why our patient did not develop PCD, at the time of the initial diagnosis of HL remain unclear. At that time the tumor burden was significantly higher (Ann Arbor stage III B) than at the relapse 12 years later (stage 1 A). This observation favors the idea that the immune response was more aggressive at the relapse but at the same time more effective in controlling the cancer [6] and is supported by the finding that in SCLC the presence of anti-Hu antibodies correlated with limited tumor stage, response to chemotherapy and longer survival [7].

The pathological hallmark of PCD is a widespread loss of Purkinje cells of the cerebellar cortex. The target antigen of the Tr-antibodies has been identified as DNER (Delta/notch-like epidermal growth factor-related receptor) [8, 9]. This protein is strongly expressed in the dendrites of Purkinje cells and is important for the function of these neurons. Unlike other onconeuronal antibodies in PCD (e.g., anti-Hu in SCLC, anti-Yo in ovarian or breast cancer), there is no evidence of immune activity to anti-Tr/DNER antibodies in malignant lymphoma tissue suggesting, that the pathological immune response is not triggered by tumor expression itself but likely from a more complex immune dysregulation caused by the tumor [8]. In addition to anti-Tr antibodies, mGluR1 and mGluR5 antibodies can be associated with HL. Both these antibodies were negative in our patient. mGluR1 antibodies has been described in two patients with PCD 2 and 9 years after HL [10]. In contrast to anti-Tr and mGluR1 antibodies, mGluR5 antibodies associated with HL causes a subacute syndrome with confusion and neuropsychiatric symptoms [11].

PCD related to the Tr/DNER antibodies does not respond as well to treatment as other disorders associated to antibodies against cell surface antigens does. Various treatment strategies (high-dose steroids, IVIG and plasmapheresis given a single therapy or in combination) have been reported. Our patient had benefit of serial IVIG treatments in combination with promptly cancer treatment.

In our patient there was a diagnostic delay of 3–4 weeks before a correct diagnosis was made and the case demonstrates three important diagnostic pitfalls: (1) Initially, the symptoms were wrongly interpreted as a stroke, (2) HL is normally considered as cured after 5 years without a relapse. The FDG-PET/CT finding could easily had been misdiagnosed as a Warthin tumor which is a benign condition so that further investigation not was initiated and the relapse had remained undiagnosed and (3) lack of attention of the oncological history at the first admission. This highlights that the presence of a paraneoplastic syndrome should be considered even in patients with very long cancer remission so that treatment can be initiated as early as possible. The increasing high cure rate and low relapse rate in many cancers does not exclude the possibility that a PNS can be the first and only sign of a relapse.

## Conclusions

Late relapse in HL is very rare. If it occurs it presents as a symptomatic lymphadenopathy. Presentation of a very late relapse of HL as a PCD 12 years after a complete remission from first HL has not previously been reported. PCD can in rare cases mimic a stroke within the posterior cerebrovascular circulation. If MR imaging in severe acute/subacute cerebellar syndrome is normal further investigation is mandatory to rule out a PNS, particular in patients with a previous history of cancer. In the future, the role of PNS in cancer relapse must be elucidated in larger studies.

## Data Availability

Data sharing is not applicable to this article as no datasets were generated or analyzed during the current study.

## Abbreviations

FDG-PET/CT: ^18^F-FDG: Positron emission tomography/contrast enhanced computed tomography
Anti-Tr/DNER: anti-Tr: Trotter (named after JL Trotter who initially described the antibody), Delta/notch-like epidermal growth factor-related receptor
CSF: Cerebrospinal fluid
HL: Hodgkin Lymphoma
i.v.: intravenous
IVIG: Intravenous immunoglobulin
MRI-scan: Magnetic resonance imaging scanning
mGluR1: metabotrophic glutamate receptor 1
mGluR5: metabotrophic glutamate receptor 5
PCD: Paraneoplastic cerebellar degeneration
PNS: paraneoplastic neurological syndrome
SCLC: Small cell lung cancer
